# Discovery of a New Compound, Erinacerin W, from the Mycelia of *Hericium erinaceus*, with Immunomodulatory and Neuroprotective Effects

**DOI:** 10.3390/molecules29040812

**Published:** 2024-02-09

**Authors:** Jing-Yi Lin, Yen-Po Chen, Ting-Wei Lin, Tsung-Ju Li, Yu-Wen Chen, I-Chen Li, Chin-Chu Chen

**Affiliations:** 1 Biotech Research Institute, Grape King Bio Ltd., Long Tan Dist., Taoyuan City 325, Taiwan; jingyi.lin@grapeking.com.tw (J.-Y.L.); yp.chen@grapeking.com.tw (Y.-P.C.); tingwei.lin@grapeking.com.tw (T.-W.L.); tsungju.li@grapeking.com.tw (T.-J.L.); annie.chen@grapeking.com.tw (Y.-W.C.); 2 Institute of Food Science and Technology, National Taiwan University, Taipei City 106, Taiwan; 3 Department of Food Science, Nutrition and Nutraceutical Biotechnology, Shih Chien University, Taipei City 104, Taiwan; 4 Department of Bioscience Technology, Chung Yuan Christian University, Zhong-Li Dist., Taoyuan City 320, Taiwan

**Keywords:** novel compound, erinacerin W, *Hericium erinaceus* mycelia, NMR, neuroprotection, immunomodulatory

## Abstract

One new compound with an isoindolinone skeleton, along with erinacines A, C, and S, was isolated from the mycelia of *Hericium erinaceus*, an edible fungus with a long history of use in traditional Chinese medicine. Based on analysis of MS and NMR spectral data, the structure of the compound was identified as (2E,6E)-8-(2-(1-carboxy-3-methylbutyl)-4,6-dihydroxy-1-oxoisoindolin-5-yl)-2,6-dimethylocta-2,6-dienoic acid. In light of this discovery, we have given this compound the name erinacerin W. Using a co-culture in vitro LPS-activated BV2 microglia-induced SH-SY5Y neuroinflammation model, the results showed that erinacerin W demonstrated protection against the LPS-activated BV-2 cell-induced overexpression of IL-6, IL-1β, and TNF-α on SH-SY5Y cells. This finding may provide potential therapeutic approaches for central nervous disorders.

## 1. Introduction

Neurodegenerative diseases are slow processes that cause irreversible damage to specific areas of the brain, resulting in reduced memory and cognitive function loss. The most common neurodegenerative disorders are Alzheimer’s disease (AD) and Parkinson’s disease (PD) [1]. With the rapid increase in the aging population, it can be expected that neurodegenerative diseases will impose a large economic burden on global medical care, the economy, families, and society in the future. Given the lack of curative treatments for neurodegenerative diseases, there has been a growing interest in exploring the potential of natural products for neuroprotection in recent years [2].

One such natural product is *Hericium erinaceus*, a medicinal and edible fungus with a rich and extensive history of use in traditional medicine and cuisine [3]. Several medicinal properties of *H. erinaceus* have been studied, including its immune-modulatory, antioxidant, cholesterol-lowering, blood sugar-lowering, antimicrobial, and anticancer activities [4,5,6]. These effects are attributed to its bioactive components, such as erinacines, hericenones, hericerins, erinacerins, erinaceolactones, alkaloids, steroids, polysaccharides, and glycoproteins [7]. However, it is important to note that the expression of bioactive secondary metabolites in the mycelia and fruiting bodies of the fungus can vary significantly due to their distinct structures [8]. While it is possible to synthesize some of these compounds, the synthesis of erinacine A, which has a complex structure, requires a lengthy and challenging process with low yield [9]. Apart from erinacines B and E, which also require complex synthesis processes, there has been no reported synthesis of other erinacines or erinacerin series compounds. Therefore, through a controlled manipulation of *H. erinaceus* mycelia in a bioreactor, such as adjusting the temperature, aeration speed, and pH, it is possible to elicit the production of the active compounds known as erinacines and erinacerins [10,11]. Consequently, there has been a remarkable increase in research reports focusing on the potential applications and safety of metabolite-enriched *H. erinaceus* mycelia over the past decade [12].

## 2. Results and Discussion

**Figure 1:**
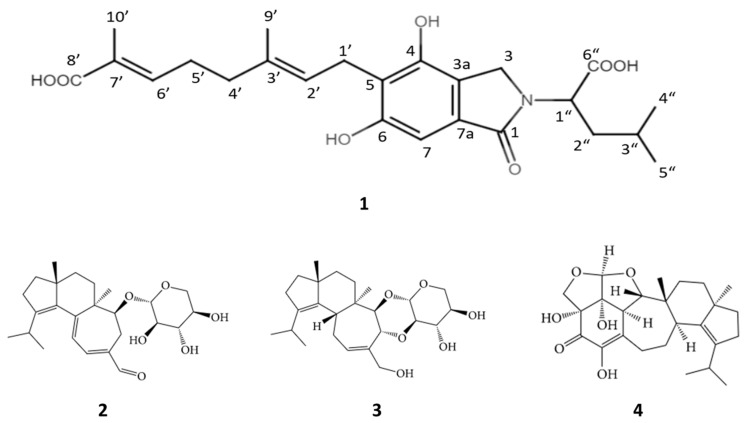
The molecular structures of compounds 1–4 investigated in this study. **1**: erinacerin W, (2E,6E)-8-(2-(1-carboxy-3-methylbutyl)-4,6-dihydroxy-1-oxoisoindolin-5-yl)-2,6-dimethylocta-2,6-dienoic acid; **2**: erinacine A, 3a,5a-dimethyl-1-(propan-2-yl)-6-[(3,4,5-trihydroxyoxan-2-yl)oxy]-2H,3H,3aH,4H,5H,5aH,6H,7H-cyclohepta[e]indene-8-carbaldehyde; **3**: erinacine C, (1R,2R,5R,10R,14R,16R,17S,18R,21S)-13-(hydroxymethyl)-2,5-dimethyl-8-propan-2-yl-15,20,22-trioxapentacyclo [12.8.0.02,10.05,9.016,21]docosa-8,12-diene-17,18-diol; **4**: erinacine S, (2aR,6aR,9aR,11aR,11bS,12aS,12bR,12cR)-2,2a,6,6a,8,9,9a,10,11,11a,11b,12a,12b,12c-Tetradecahydro-2a,4,12b-trihydroxy-9a,11a-dimethyl-7-(1-methylethyl)furo [2,3,4-cd]indeno [5′,4′:4,5]cyclohept [1,2,3-hi]isobenzofuran-3(5H)-one.

One new compound with an isoindolinone skeleton (**1**), along with erinacines A (**2**), C (**3**), and S (**4**), was isolated from the mycelia of *Hericium erinaceus*. Based on its unique skeleton structure, this new compound was categorized as a member of the erinacerin series and named erinacerin W (**1**) (Figure 1). Erinacerin W (**1**) was obtained as a pale-yellow clear oil with no specific odor. It has a molecular formula of C_24_H_31_NO_7_ and a molecular weight ion of *m/z* 444.202 [M − H] −, determined by LC-QTOF MS (Santa Clara, CA, USA).

The ^1^H and ^13^C in the CD_3_OD spectra of **1** were obtained using a Bruker 400 MHz spectrometer (Bruker BioSpin, Rheinstetten, Germany) and are shown in Table 1.

**Figure 2:**
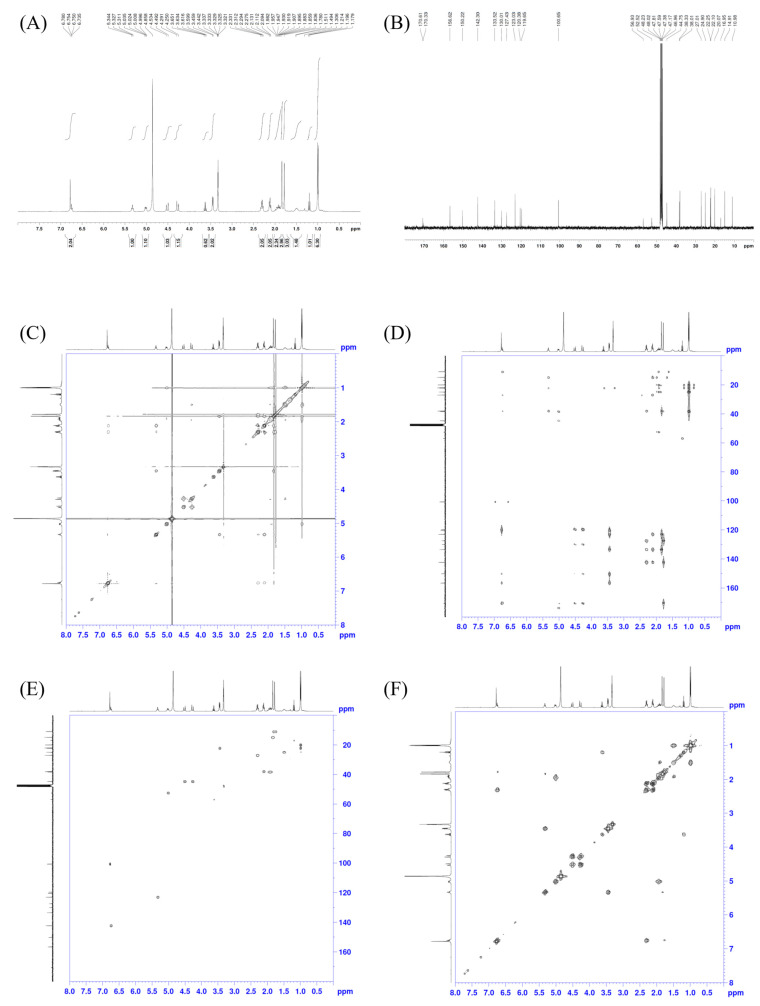
The (**A**) 1H, (**B**) 13C, (**C**) NOESY, (**D**) HMBC, (**E**) HMQC, and (**F**) COSY NMR spectral data of **1**.

The ^1^H, ^13^C, NOESY, COSY, HMQC, and HMBC NMR spectral data (Figure 2) of compound **1** revealed the presence of various functional groups and carbon shifts. Firstly, two olefinic methyl groups were identified, with chemical shifts of δH 1.84 (3H, s) and 1.78 (3H, s), and corresponding carbon shifts of δC 14.9 and 11.0. Additionally, five methylenes were observed, with proton chemical shifts of δH 4.52 (1H, d, J = 16.8 Hz), 4.27 (1H, d, J = 16.8 Hz), 3.45 (2H, d, J = 6.8 Hz), 2.11 (2H, t, J = 7.6 Hz), 2.30 (2H, q, J = 7.6 Hz), and 1.95 (2H, m), and corresponding carbon shifts of δC 44.8, 22.3, 38.0, 27.0, and 38.3. Furthermore, an aromatic proton with a chemical shift of δH 6.78 (1H, s) and a corresponding carbon shift of δC 100.7 was observed. The NMR data also indicated the presence of two trisubstituted olefins, with proton chemical shifts of δH 5.33 (1H, t, J = 6.8 Hz) and 6.75 (1H, t, J = 7.6 Hz), and carbon shifts of δC 123.0 (=CH), 133.5, 142.3 (=CH), and 127.4. Moreover, five tertiary aromatic carbons were detected, with carbon shifts of δC 119.7, 120.4, 130.0, 150.4, and 156.6. Additionally, three carbonyl groups were identified, with carbon shifts of δC 170.6, 170.6, and 170.3. Lastly, two methyl carbons were observed, with carbon shifts of δC 20.1 and 22.1. These data collectively suggest that compound **1** possesses an isoindolinone skeleton. Further connectivity was established by a long-range HMBC experiment, and the correlations are shown in Table 1.

**Figure 3:**
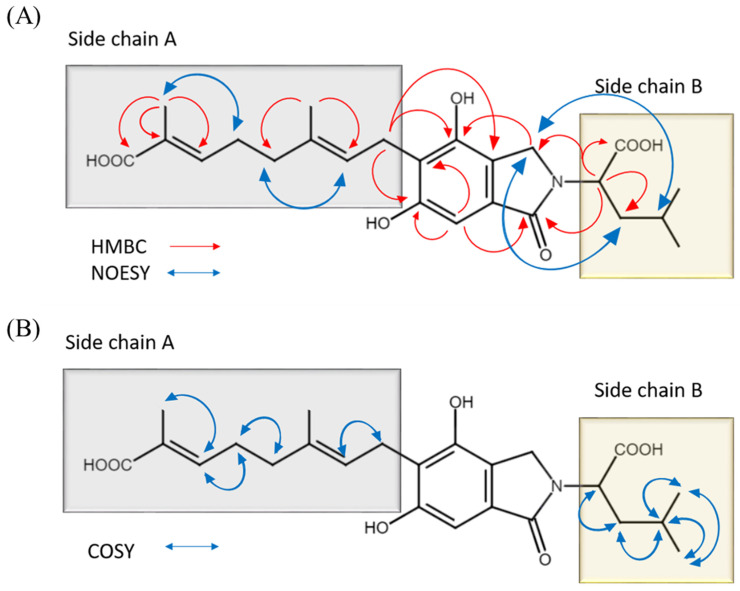
(**A**) Key HMBC, NOESY, and (**B**) COSY correlations of **1**.

The HMBC correlations were observed between H2-3 and C-1, C-3a, C-4, and C-7a; between H-7 and C-1, C-3a, C-5, C-4, and C-6; between H2-1′ and C-2′, C-3′, C-4, C-5, C-6, and C-3a; between H2-5′ and C-3′, C-4′, C-6′, and C-7′; between H3-9′ and C-2′ and C-4′; between H3-10′ and C-6′, C-7′, and C-8′; between H-1″ and C-1, C-3, C-2″, and C-6″; between H2-2″ and C-1″, C-3″, C-4″, and C-5″; and between H3-4″ and C-2″, C-3″, and C-5″. Based on the above analysis, H-1″ had a 3J coupling with C-3. Side chain B connects the core of structure 1 through N. Similarly, H-1′ had a 3J coupling with C-4 and C-6. Therefore, side chain A connects the core of structure 1 by C-5, as shown in Figure 3A.

Multiple correlations were observed in the NOESY analysis, including a correlation between H-2′ and H2-4′ and another correlation between H2-5′ and H3-10′, as well as correlations between H2-2″ and H2-3 and between H2-3″ and H2-3 (Figure 3A). COSY correlations were observed between H2-1′ and H-2′, between H2-4′ and H2-5′, between H2-5′ and H-6′, between H-1″ and H2-2″, between H2-2″ and H-3″, and between H-3″ and H3-4″, H3-5″ (Table 1 and Figure 3B). Based on the analysis conducted, the structure of compound 1 was identified as (2E,6E)-8-(2-(1-carboxy-3-methylbutyl)-4,6-dihydroxy-1-oxoisoindolin-5-yl)-2,6-dimethylocta-2,6-dienoic acid.

Among erinacerins, erinacerin F and compound **1** share similar structural features, as they both possess two hydroxy groups, two carboxyl groups, and two carbonyl groups [13]. These functional groups contribute to the high polarity of their overall structures. However, the main point of differentiation between erinacerin F and compound **1** lies in the side chain at the 3″ position on the N atom. In erinacerin F, the side chain consists of an isoleucine, while compound **1** presents a leucine in this position. This slight alteration in the side-chain configuration leads to a distinct structural variation between the two compounds.

Based on their structures, it can be deduced that the polarities of erinacerin F and compound **1** are similar. However, to completely understand the effect of this structural distinction on their chemical properties and potential biological activities. It will be important to determine the absolute configuration and specific rotation of compound **1**, as chiral molecules have greatly influenced the development of new drugs. Therefore, for future research, all of these factors should be taken into consideration.

Natural products offer an opportunity to explore new compounds that can be developed into drugs due to their diverse chemical structures. Currently, a total of 22 erinacerins have been discovered, representing a rich pool of compounds for further investigation. While erinacerins A, C, M-N, and U-V are still undergoing rigorous exploration and in-depth investigation to uncover their full range of potential applications [14,15,16], previous research has shown that erinacerin B could reduce LPS-induced nitric oxide (NO) and prostaglandin E2 (PGE2) production in RAW 264.7 macrophage cells in a concentration-dependent manner, thus exhibiting anti-inflammatory properties [17]. Moreover, erinacerins D-L act as α-glucosidase inhibitors, with IC_50_ values in the range of 12.8 to 145.1 μM [13]. Additionally, erinacerins O-P have emerged as promising candidates for potential glioma inhibitors, as they have demonstrated the ability to induce apoptosis in U87 cells through the Bax/Capase-2 pathway [18]. Furthermore, erinacerins Q-T have exhibited notable inhibitory activity against protein tyrosine phosphatase-1B (PTP-1B), with IC_50_ values ranging from 24.9 to 42.1 μM [19]. In this study, for the first time, erinacerin W demonstrated anti-neural inflammation and the potential to inhibit the onset and progression of neurodegenerative diseases, distinguishing it from other erinacerin series.

**Figure 4:**
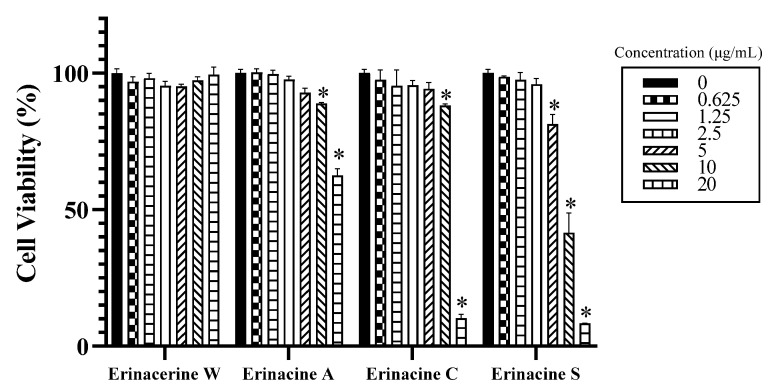
Concentration–effect curves of compounds **1**–**4** in SH-SY5Y cells were assessed after 24 h of exposure using the MTT assay. The results were expressed as a percentage of cell viability relative to the untreated controls and represented as the mean  ±  standard deviation (SD) of three independent experiments. * indicates a significant difference compared to the control group (*p* < 0.05).

Co-culture modules using SH-SY5Y neuroblastoma and BV2 microglia cells have been successfully utilized to study the harmful effects of microglial activation in neurodegenerative diseases [20]. To examine the potential impacts of compounds **1**–**4** on LPS-activated microglia-mediated cell viability, we investigated the co-culture of BV-2 microglia with SH-SY5Y neurons. After incubating SH-SY5Y cells with a range of concentrations (from 0 to 20 µg of compounds **1**–**4**) for 24 h, it was observed that compound **1** had no cytotoxic effect up to 20 µg/mL, while compounds **1** and **2** above 5 µg/mL and compound **3** above 2.5 µg/mL reduced cell viability by approximately 10% (Figure 4). Therefore, non-toxic doses were selected for further experiments.

**Figure 5:**
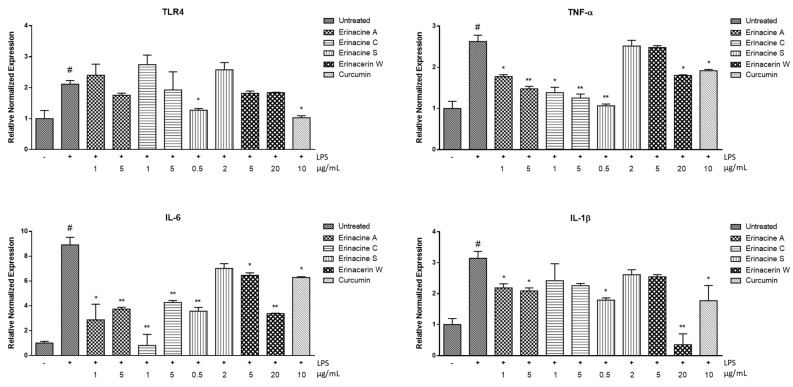
Effects of compounds **1–4** and curcumin (positive control) on the expression of TLR4, IL-6, IL-1β, and TNF-α in LPS-stimulated BV2 microglia-mediated SH-SY5Y cells. The mRNA expression levels were calculated relative to a GAPDH control using standard curves. # Significant difference in comparison with the untreated group (*p* < 0.05). * Significant difference in comparison with LPS-induced group (*p* < 0.05). The use of double asterisks (**) indicates a significant difference with a *p-*value of less than 0.01. Results were expressed as mean  ±  standard deviation (SD).

Consistent with a previous study [21], the mRNA levels of TLR4, IL-6, IL-1β, and TNF-α significantly increased in LPS-stimulated BV2 microglia-mediated SH-SY5Y cells, as shown in Figure 5 (*p* < 0.05). The activation of these signaling proteins, also known as a cytokine storm, not only plays important roles in normal bodily functions and innate immunity [22], but is also associated with neurological diseases [23]. In this study, compounds **2** and **3** effectively reduced proinflammatory signaling proteins, such as IL-6 and TNF-α, induced by LPS, which is consistent with earlier research [24,25]. Additionally, exposure to a low dose, rather than a high dose, of compound **4** showed a decrease in the neuroinflammatory response, potentially promoting cellular repair and protection. Furthermore, for the first time, compound **1,** at a concentration of 20 µg/mL, demonstrated protection against the excessive expression of IL-6, IL-1β, and TNF-α in SH-SY5Y cells induced by LPS-activated BV-2 cells. Previous studies have shown that LPS-induced inflammation in BV-2 microglia cells increases TLR4 expression and activates the NF-κB and MAPKs pathways, leading to the production of TNF-α, IL-6, and IL-1β [26]. In line with this, compounds **1**–**4** may decrease the production of TNF-α, IL-6, and IL-1β, possibly involving pathways such as NF-κB or MAPK. The significant finding of this study is that erinacerin W demonstrated low cell toxicity while effectively mitigating neural inflammation. This indicates its possible use in slowing down, or potentially even halting, the progression of neurodegenerative diseases. However, more studies are needed for further clarification. Overall, considering the importance of the cytokine storm in neurodegeneration, compounds **1**–**4** may indirectly have protective potential by regulating microglia-mediated inflammatory processes, thus offering promising therapeutic approaches for CNS disorders.

## 3. Experimental Section

### 3.1. General Experimental Procedures

^1^H, ^13^C, and 2D NMR spectra were obtained with a Bruker 400 MHz spectrometer (Bruker BioSpin, Rheinstetten, Germany). The Agilent 6546 LC/Q-TOF system (Santa Clara, CA, USA) was used to measure the MS data. Solvents such as methanol, ethyl acetate, and n-hexane, used for extraction and chromatographic separation, were analytical-grade (Merck, Darmstadt, Germany). Silica gel 60 (Merck, Darmstadt, Germany) and Cosmosil C_18_-OPN (Nacalai Tesque, Kyoto, Japan) were used for column chromatography.

### 3.2. Material Preparation

*Hericium erinaceus* (BCRC 35669) was purchased from the Bioresources Collection, Research, and Development Institute in Hsinchu, Taiwan. A fresh *H. erinaceus* mycelium block (1 cm^3^) was added to a 2 L Erlenmeyer flask containing 1.3 L of a modified medium. The modified medium consisted of 0.25% yeast extract, 4.5% glucose, 0.5% soybean powder, 0.25% peptone, and 0.05% MgSO_4_, with the pH adjusted to 4.5. The entire medium was then incubated at 26 °C on a 120 rpm shaker for 5 days. Following this, the fermentation process was scaled up from a 2 L shake flask to 500 L and continued for 5 days. The mycelia grown on the complete medium were harvested, freeze-dried, and ground into a fine powder.

### 3.3. Extraction and Isolation

**Figure 6:**
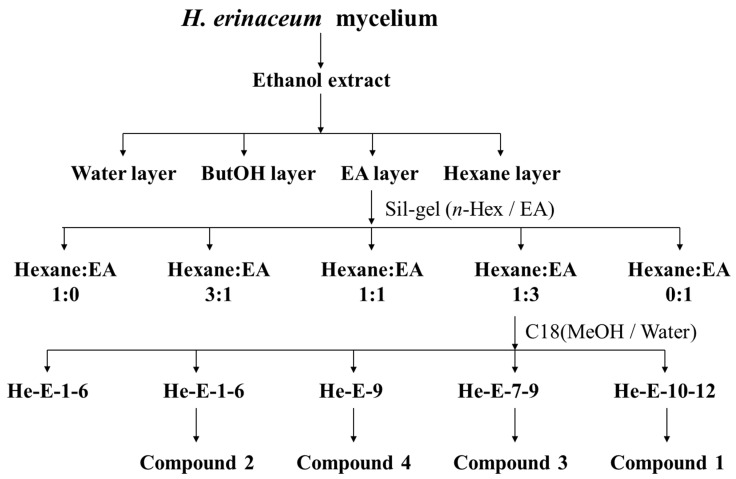
Schematic diagram of the extraction and isolation of compounds **1**–**4** from *H. erinaceus* mycelia.

A schematic diagram of the extraction and isolation of compounds from *H. erinaceus* mycelia is shown in Figure 6.

The freeze-dried mycelia of *H. erinaceus* (250 g) were refluxed with 95% ethanol. The ethanol solution was concentrated under vacuum to produce a brown extract, which was then divided into a water layer, ButOH, EA, and a Hexane layer using a H_2_O–EA (1:1) partitioning. The structure of the known compounds, erinacines A (**2**), C (**3**), and S (**4**), was identified by correlating the experimental NMR data with values published in previous studies [27,28]. For an additional novel compound, the EA layer was purified by chromatography on a silica gel (70–230 mesh) column, using a gradient system of n-hexane–EA (1:0 to 0:1), resulting in five fractions. Fraction 4 (n-hexane–EA = 1:3 eluate) was further separated using HPLC Fraction Collectors (reversed-phase COSMOSIL 5C_18_-AR-II column, Nacalai Tesque, Kyoto, Japan) and eluted with a gradient system of H_2_O–acetonitrile (4:1 → 1:4) to isolate compound **1**, a novel isoindolinone compound (13 mg).

#### Erinacerin W (**1**)

C_24_H_31_NO_7_; yellow oil; ^1^H and ^13^C NMR spectral data in CD_3_OD (see Table 1); calcd monoisotopic mass: *m/z* 444.2027 [M – H] −.

### 3.4. Co-Culture of BV-2 Microglia and SH-SY5Y Neurons

The human neuroblastoma cell line SH-SY5Y (ATCC #CRL-2266) was cultured in a 6-well plate (1 × 10^5^ cells/mL), while BV-2 cells (ICIC #ATL03001) at 1  ×  10^5^ cells/mL were placed on cell culture inserts (pore size: 0.4 μm; Corning, New York, NY, USA). The BV-2 cells were stimulated with LPS (100 ng/mL) for 24 h, and the SH-SY5Y cells were treated with compounds **1**–**4** (concentrations determined by cell viability assay) for 24 h before being co-cultured together for another 24 h in DMEM containing 10% (*v*/*v*) heat-inactivated FBS, 100 U/mL of penicillin, and 100 mg/mL of streptomycin at 37 °C in a humidified incubator with 5% CO_2_, as previously described [29]. Untreated cells were used as controls, while cells incubated with curcumin served as the positive control [30].

### 3.5. Total RNA Isolation and Quantitative PCR (qPCR) Analysis

After treating the SH-SY5Y cells with the specified agents, they were collected and lysed to assess the expression of TLR4, IL-6, IL-1β, and TNF-α. Total RNA was isolated using an RNA purification kit (Thermo Fisher Scientific, Waltham, MA, USA) following the manufacturer’s instructions. The isolated RNA (1.0 μg) was then reverse-transcribed using the iScript cDNA Synthesis Kit (Bio-Rad, Hercules, CA, USA) to generate cDNA. The relative mRNA expression level was normalized to GAPDH expression, and the ∆∆Ct method was used for quantification.

### 3.6. Statistical Analysis

All the graphs, calculations, and statistical analyses were performed using the GraphPad Prism software, version 8.0 (GraphPad Software, San Diego, CA, USA). The comparison of means between different groups of numerical variables was performed using a one-way ANOVA. A *p* value less than 0.05 (*p* <  0.05) was considered as statistically significant.

## 4. Conclusions

In conclusion, the newly isolated compound, erinacerin W, exhibited protective effects against inflammation-associated neurotoxicity. Furthermore, erinacines A, C, and S also showed potential in reducing proinflammatory cytokines. These findings suggest that these compounds may have therapeutic potential in the treatment of neurodegenerative diseases by regulating microglia-mediated inflammatory processes. Further research is needed to explore the full potential and mechanisms of action of these compounds and to develop effective therapies for CNS disorders. 

## Figures and Tables

**Table 1:** ^1^H and ^13^C NMR spectroscopic data for erinacerin W (**1**).

Position	δ_C_, Type	δ_H_ (*J* in Hz)	HMBC	NOESY	COSY
1	170.6, CH				
2					
3	44.8, CH_2_	4.52 d (16.8); 4.27 d (16.8)	3 a, 7 a, 4, 1	2″, 3″	
3 a	119.7, C				
4	150.4, C				
5	120.4, C				
6	156.6, C				
7	100.7, CH	6.78 s	3 a, 5, 4, 6, 1	2′, 4′, 5′	
7 a	130.0, C				
1′	22.3, CH_2_	3.45 d (6.8)	3 a, 5, 2′, 3′, 4, 6		2′
2′	123.0, CH	5.33 t (6.8)	1′, 4′, 9′	1′, 4′, 5′, 6′	1′
3′	133.5, C				
4′	38.0, CH_2_	2.11 t (7.6)	2′, 3′, 5′, 6′	7, 2′, 5′, 6′, 10′	5′
5′	27.0, CH_2_	2.30 q (7.6)	3′, 4′, 6′, 7′	2′, 4′, 6′, 7, 10′	4′, 6′
6′	142.3, CH	6.75 t (7.6)	4′, 5′, 8′, 10′	2′,4′, 5′, 9′	5′, 10′
7′	127.4, C				
8′	170.3, C				
9′	14.9, CH_3_	1.84 s	2′, 4′	4′, 5′, 6′	
10′	11.0, CH_3_	1.78 s	6′, 7′, 8′	4′, 5′	6′
1″	52.5, CH	5.01 dd (4.4, 10.8)	1, 3, 2″, 6″	2″, 4″, 5″,	2″
2″	38.3, CH_2_	1.95 m	1″, 3″, 4″, 5″	1″, 3, 7, 6′,	1″, 3″
3″	24.9, CH	1.5 m		3, 2″, 4″, 5″	2″, 4″, 5″
4″	20.1, CH_3_	1.00 d (7.6)	2″, 3″, 5″	1″, 2″, 3″, 5″	3″, 5″
5″	22.1, CH_3_	1.00 d (7.6)	2″, 3″, 4″	1″, 2″, 3″ 4″	3″, 4″
6″	170.6, C				

## Data Availability

Data are contained within the article.
